# Routine Intraoperative ICG Perfusion Assessment and Anastomotic Leak After Esophagectomy: A Before–After Cohort Study

**DOI:** 10.3390/jcm15176827

**Published:** 2026-09-03

**Authors:** Luca Galassi, Alberto Aiolfi, Emanuele Morandi, Francesco Cammarata, Carlo Banfi, Gianluca Bonitta, Marta Cavalli, Giampiero Campanelli, Luigi Bonavina, Davide Bona

**Affiliations:** 1 Department of Biomedical Sciences for Health, Postgraduate School of Vascular and Endovascular Surgery, University of Milan, Via Festa del Perdono 7, 20122 Milan, Italy; luca.galassi@unimi.it; 2 Division of General Surgery, Department of Biomedical Science for Health, IRCCS Ospedale Galeazzi–Sant’Ambrogio, University of Milan, Via Cristina Belgioioso 173, 20157 Milan, Italy; emanuele.morandi@unimi.it (E.M.); francesco.cammarata@grupposandonadonato.it (F.C.); gianluca.bonitta@grupposandonadonato.it (G.B.); marta.cavalli@grupposandonadonato.it (M.C.); giampiero.campanelli@grupposandonadonato.it (G.C.); davide.bona@unimi.it (D.B.); 3 Department of Cardiovascular and Thoracic Surgery and Lung Transplantation, Centre Hospitalier Universitaire UCL Namur (Godinne Site), Université Catholique de Louvain, 1348 Yvoir, Belgium; carlo.banfi@chuuclnamur.uclouvain.be; 4 Geneva Hemodynamic Research Group, Faculty of Medicine, University of Geneva, 1205 Geneva, Switzerland; 5 Department of Pharmacy, Health and Nutrition Sciences, Azienda Ospedaliera di Cosenza, Division of General and Foregut Surgery, University of Calabria, 87036 Cosenza, Italy; luigi.bonavina@unimi.it

**Keywords:** esophagectomy, anastomotic leak, indocyanine green, fluorescence angiography, gastric conduit, perfusion, esophageal cancer

## Abstract

**Background:** Anastomotic leak (AL) is a major complication after esophagectomy, and gastric conduit perfusion is considered a key determinant of anastomotic integrity. Indocyanine green (ICG) fluorescence angiography is increasingly used for intraoperative perfusion assessment, although its clinical effect remains uncertain. We evaluated whether routine implementation of ICG assessment was associated with a lower incidence of AL compared with a historical no-ICG cohort, and explored intraoperative factors potentially associated with AL. **Methods:** Single-center before–after cohort study of consecutive adults undergoing Ivor-Lewis esophagectomy for cancer between January 2023 and December 2025, using a prospectively maintained database. ICG entered routine practice in March 2025, defining a historical no-ICG cohort and an ICG cohort. ICG (2 mL of a 25 mg/10 mL solution) was administered at three intraoperative timepoints; time to fluorescence (TTF), arcade–conduit fluorescence pattern, arterial blood gas values, and hemodynamic parameters were recorded. AL was defined according to Esophagectomy Complications Consensus Group criteria. **Results:** Sixty patients were included (ICG, n = 17; no-ICG, n = 43). AL occurred in 4/17 (23.5%) versus 5/43 (11.6%) patients (odds ratio 2.34, 95% CI 0.54–10.05; *p* = 0.256). Overall postoperative morbidity, conduit necrosis, pulmonary and infectious complications, reintubation, and 90-day mortality did not differ; a single death occurred, in the no-ICG cohort. All TTF values were below 60 s (range 23–46 s) and did not differ between patients with and without AL, nor did the fluorescence pattern; ICG never modified the planned anastomotic site or prompted additional conduit resection. Exploratory within-ICG analyses showed lower thoracic-phase PaO_2_ and PaCO_2_ and higher pH in patients who developed AL. **Conclusions:** Routine qualitative ICG assessment of gastric conduit perfusion was feasible; however, this small before–after cohort was insufficient to determine its effect on AL. Larger studies using standardized quantitative fluorescence assessment are required.

## 1. Introduction

Esophageal cancer represents one of the most prevalent malignancies worldwide and remains a major contributor to cancer-related mortality. Squamous cell carcinoma and adenocarcinoma constitute the predominant histological subtypes, together accounting for the vast majority of cases [1,2,3]. In patients with resectable or locally advanced disease, esophagectomy, frequently integrated with neoadjuvant chemoradiotherapy or perioperative chemotherapy, remains a central component of curative-intent management [4,5,6].

Notwithstanding advances in minimally invasive surgical techniques, perioperative care pathways, and the centralization of esophageal surgery [7], esophagectomy continues to be associated with considerable morbidity and mortality. In contemporary international benchmarking series based on standardized definitions, overall complication rates approach 60% and AL still occurs in more than 10% of patients [8,9]. Among postoperative complications, anastomotic leak (AL) is particularly consequential, as it represents a major determinant of postoperative morbidity and mortality and may adversely influence quality of life, length of hospital stay, and long-term survival [10,11,12,13,14]. The Esophagectomy Complications Consensus Group (ECCG) defines AL as a full-thickness gastrointestinal defect involving the esophagus, anastomosis, staple line, or conduit, irrespective of clinical presentation or diagnostic modality [10].

Although the pathogenesis of AL is multifactorial, gastric conduit perfusion is widely considered a principal determinant of anastomotic integrity, because conduit tubularization substantially reduces gastric arterial inflow, leaving the right gastroepiploic artery as the dominant vascular supply [15,16]. Patient-related factors, including active or previous smoking, may further compromise tissue perfusion and increase the risk of AL [11,17]. Conversely, strategies intended to augment conduit perfusion, most notably gastric ischemic conditioning, have been associated in meta-analyses with reduced leak rates and lower leak severity [18,19,20]. In accordance with the central role of perfusion, reduced conduit microvessel density and a shorter right gastroepiploic artery have also been associated with a higher incidence of AL [15,21].

Historically, intraoperative evaluation of gastric conduit viability relied on subjective clinical judgment or on techniques such as laser Doppler flowmetry, tissue reflectance spectroscopy, and gastric mucosal pCO_2_ monitoring, whose reproducibility and correlation with AL proved inconsistent [22,23,24,25]. Indocyanine green (ICG) fluorescence angiography has increasingly been adopted as a practical, real-time intraoperative modality for evaluating gastric conduit perfusion and informing selection of the anastomotic site, an application first reported in esophageal reconstructive surgery more than a decade ago [26]. Delayed fluorescence has been proposed as a surrogate marker of hypoperfusion, as exemplified by the so-called 90-s rule, and ICG-guided resection of poorly perfused conduit tissue has been associated with lower leak rates [27,28,29]. A recent meta-analysis suggested that ICG-based assessment may reduce AL; however, it also emphasized that heterogeneity in study design, variability in leak definitions, and potential reporting bias may lead to overestimation of its clinical effectiveness [30,31,32,33,34]. Importantly, AL may still occur despite fluorescence-guided intraoperative decision-making [35,36]. A recognized limitation of conventional ICG imaging is its qualitative and operator-dependent nature. Accordingly, quantitative software platforms, including SERGREEN, SPY-Q, FLER, and Q-ICG, as well as recently proposed randomized quantitative fluorescence thresholds, have been introduced to improve the objectivity and reproducibility of perfusion assessment [37,38,39,40,41]. In parallel, systematic reviews of fluorescence metrics and an international consensus of the European Association for Endoscopic Surgery have called for standardized acquisition, dosing, and reporting of ICG perfusion data [42,43].

We conducted a single-center before–after cohort study to evaluate whether the routine implementation of intraoperative ICG fluorescence assessment of gastric conduit perfusion was associated with a lower incidence of AL after esophagectomy. Secondary objectives included the comparison of gastric conduit necrosis and other short-term postoperative outcomes between the two cohorts. In exploratory analyses restricted to patients undergoing ICG assessment, we investigated whether fluorescence characteristics, including time to fluorescence (TTF) and synchronous versus asynchronous arcade–conduit fluorescence patterns, as well as intraoperative hemodynamic, ventilatory, and metabolic parameters, were associated with AL.

## 2. Materials and Methods

### 2.1. Study Design and Population

This single-center observational before–after cohort study was conducted at the Division of General Surgery, IRCCS Ospedale Galeazzi–Sant’Ambrogio (Milan, Italy), using retrospectively analyzed data from a prospectively maintained institutional database. All procedures were performed by two surgeons with experience in upper gastrointestinal surgery (A.A. and D.B.). Consecutive adult patients (≥18 years) who underwent transthoracic Ivor-Lewis esophagectomy for esophageal adenocarcinoma between January 2023 and December 2025 were included.

**Figure 1:**
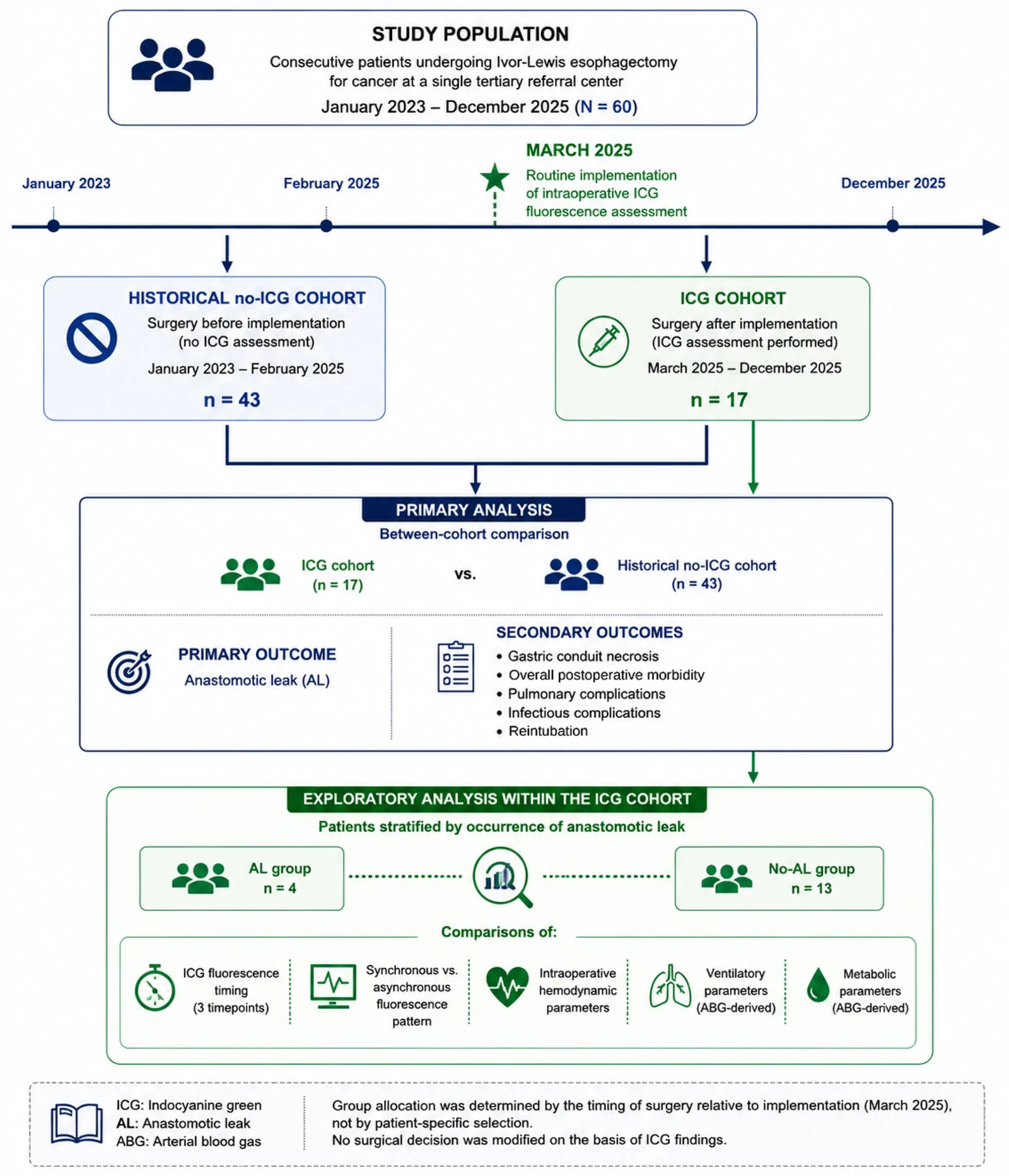
Study design and analytical framework. ICG fluorescence assessment was introduced into routine clinical practice in March 2025. Patients undergoing Ivor-Lewis esophagectomy before implementation constituted the historical no-ICG cohort (n = 43), whereas patients treated after implementation constituted the ICG cohort (n = 17). The primary analysis compared the incidence of anastomotic leak between the two temporally defined cohorts; secondary postoperative outcomes were also compared. An exploratory analysis restricted to the ICG cohort compared fluorescence dynamics and intraoperative hemodynamic, ventilatory, and metabolic parameters between patients who developed anastomotic leak (n = 4) and those who did not (n = 13). ICG, indocyanine green; AL, anastomotic leak; ABG, arterial blood gas.

Pediatric patients and patients who underwent surgery before January 2023 were excluded. ICG fluorescence assessment was introduced into routine clinical practice in March 2025 and was thereafter systematically incorporated into the intraoperative assessment of gastric conduit perfusion. No major changes in the institutional postoperative care pathway occurred during the study period, including surveillance for AL. Accordingly, the study population comprised two temporally defined cohorts: a historical no-ICG cohort, consisting of patients treated before the routine implementation of ICG, and an ICG cohort, consisting of patients treated after its implementation. Group assignment was therefore determined by the timing of surgery relative to ICG implementation rather than by patient-specific selection (Figure 1). The study is reported in accordance with the STROBE statement for observational studies [44] (Table S1).

The primary outcome was the occurrence of postoperative AL, defined according to the ECCG criteria [10]. The primary objective was to describe the implementation of routine qualitative ICG assessment of gastric conduit perfusion and to compare the incidence of AL before and after its introduction. Secondary outcomes, as prespecified in the analytical framework summarized in Figure 1, included gastric conduit necrosis, overall postoperative morbidity, pulmonary complications (including reintubation), infectious complications, and 90-day mortality. Additional exploratory analyses were performed within the ICG cohort to investigate whether fluorescence-related parameters, including TTF and synchronous versus asynchronous arcade–conduit fluorescence patterns, as well as intraoperative hemodynamic, ventilatory, and metabolic variables, were associated with the occurrence of AL.

Preoperative assessment included upper gastrointestinal endoscopy, chest radiography, contrast-enhanced computed tomography of the chest and abdomen, and a comprehensive anesthesiologic evaluation of surgical fitness. Comorbidity burden was quantified using the Charlson Comorbidity Index [45]. The study was conducted in accordance with the Declaration of Helsinki.

### 2.2. Surgical Technique and ICG Protocol

**Figure 2:**
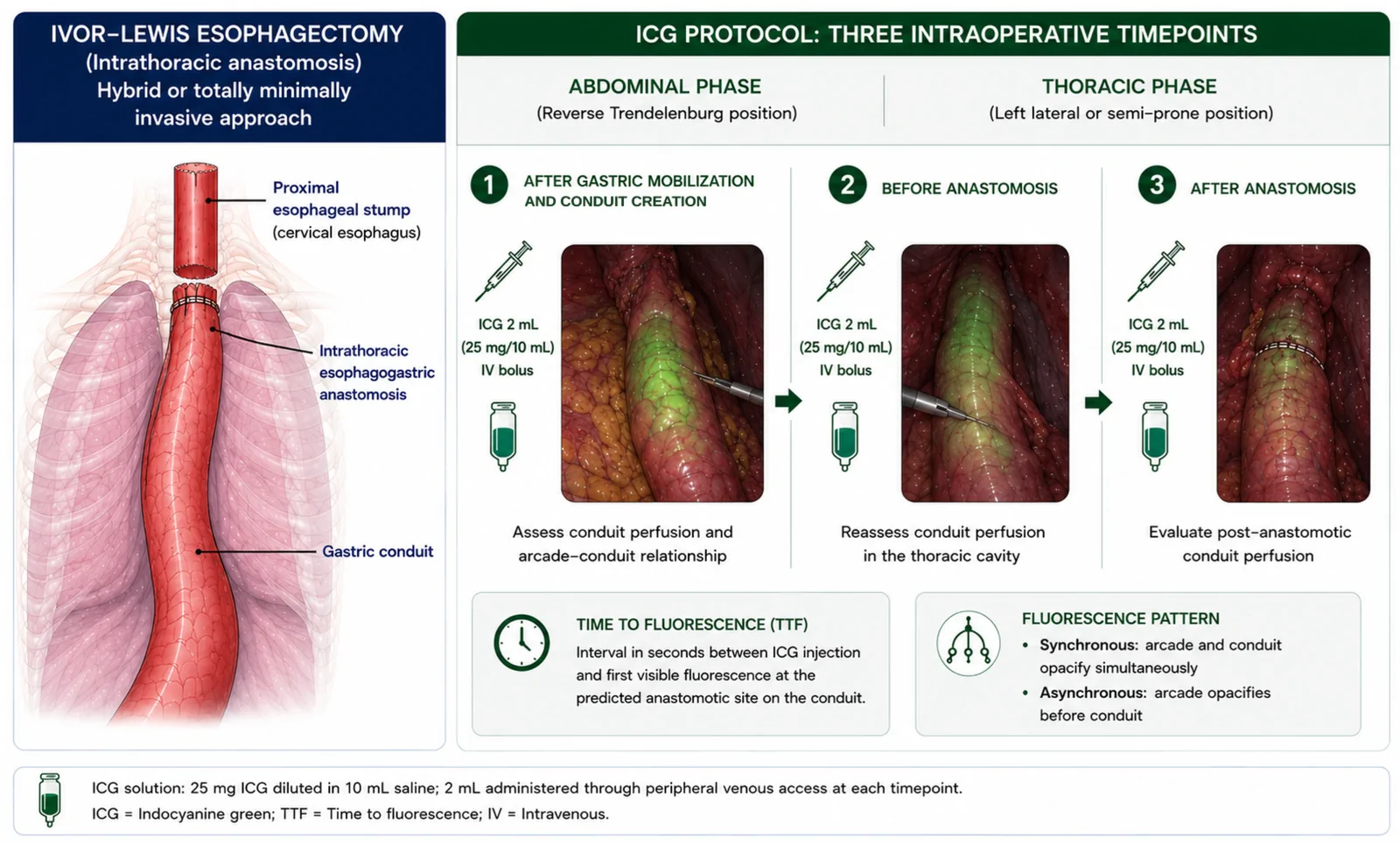
Schematic representation of Ivor-Lewis esophagectomy with intrathoracic esophagogastric anastomosis and standardized intraoperative indocyanine green (ICG) fluorescence assessment protocol. ICG was administered intravenously at three predefined operative timepoints: (1) during the abdominal phase, immediately after gastric mobilization and gastric conduit creation; (2) during the thoracic phase, before esophagogastric anastomosis; and (3) after completion of the anastomosis. At each assessment, 2 mL of ICG solution (25 mg diluted in 10 mL of saline) was administered through the same peripheral venous access, without a subsequent saline flush. The three administrations were linked to predefined operative stages rather than to fixed time intervals. Fluorescence imaging was performed using the RUBINA^®^ system (KARL STORZ) in ICG visualization mode, with operating-room lights completely switched off. Gain and exposure were automatically controlled by the imaging system and were not manually modified. Camera-to-tissue distance was not formally standardized because of the different anatomical and operative conditions at the three assessment timepoints. Time to fluorescence (TTF) was defined as the interval, in seconds, between ICG injection and the first visually detectable fluorescence at the predicted anastomotic site on the gastric conduit. TTF was assessed in real time by two observers (a medical student and an operating-room nurse), and fluorescence sequences were recorded for subsequent review. Fluorescence was additionally classified according to the relationship between opacification of the gastroepiploic arcade and the gastric conduit as synchronous, when both opacified simultaneously, or asynchronous, when the arcade opacified before the conduit. ICG, indocyanine green; TTF, time to fluorescence; IV, intravenous.

Transthoracic hybrid or totally minimally invasive Ivor-Lewis esophagectomy with intrathoracic anastomosis was performed in all included patients [46]. During the abdominal phase, patients were positioned in reverse Trendelenburg with the legs abducted, and pneumoperitoneum was established at 13 mmHg. After peritoneal lavage and dissection of the lesser omentum, the abdominal esophagus was fully mobilized, and inferior mediastinal lymphadenectomy was performed. The stomach was mobilized along the greater curvature and fundus while preserving the gastroepiploic arcade; the right and left gastric vessels were ligated and divided, and a 2.5/3 cm width gastric conduit was fashioned using a linear stapler. Immediately after tubularization, a first 2 mL dose of ICG was administered through a peripheral venous catheter placed in the patient’s arm, in order to assess conduit perfusion and to determine whether fluorescence appeared synchronously in the vascular arcade and in the conduit (Figure 2). The interval, measured in seconds, from ICG injection to fluorescence visualization at the predicted anastomotic site on the conduit was recorded (Video S1).

For the thoracic phase, one-lung ventilation was established, and patients were placed in the left lateral decubitus or semi-prone position. The thoracic phase was performed using either a minimally invasive thoracoscopic approach with five trocars or a thoracotomy through the fifth intercostal space. After ligation of the azygos vein, the thoracic esophagus was mobilized, and carinal, periesophageal, and peritracheal lymphadenectomy was performed. A second 2 mL dose of ICG was administered through the same peripheral venous catheter to evaluate conduit perfusion within the thorax before fashioning of the anastomosis. Again, the time interval (in seconds) from ICG injection to fluorescence visualization at the planned anastomotic site was recorded (Video S2). Linear-stapled anastomosis was introduced at our institution in October 2024, approximately five months before routine ICG implementation, and did not replace the circular-stapled technique; both techniques continued to be used thereafter, with the choice influenced by the operating surgeon and tumor characteristics, including tumor size. In the hybrid Ivor-Lewis technique, a circular-stapled esophagogastric anastomosis was constructed, whereas in the totally minimally invasive technique a 45 mm linear stapler was used. Subsequently, a third 2 mL ICG bolus was administered to assess post-anastomotic gastric conduit perfusion. No omental or pleural patch was used to reinforce the anastomosis in this cohort.

The ICG dilution was standardized as follows: 25 mg of ICG were diluted in 10 mL of injectable saline, and 2 mL of this solution were administered through peripheral venous access at each predefined timepoint. ICG is a tricarbocyanine dye that binds rapidly to plasma proteins and is cleared exclusively by the liver, properties that underlie its use as an intraoperative tracer of tissue perfusion [47]. TTF was defined as the interval between ICG injection and the first visible fluorescence at the predicted anastomotic site on the conduit. Synchronous or asynchronous fluorescence patterns between the gastroepiploic arcade and the conduit were also documented. Arterial blood gas analysis, including PaO_2_, PaCO_2_, pH, and lactate, was performed during both the abdominal and thoracic phases immediately before peripheral ICG injection. Postoperatively, patients were transferred to the intensive care unit. Analgesic management included intravenous paracetamol, ketorolac, and tramadol, while metoclopramide and ondansetron were administered for nausea prophylaxis or treatment. The nasogastric tube was generally removed on postoperative day 3. On postoperative day 5, a water-soluble contrast swallow study and upper gastrointestinal endoscopy were performed to evaluate anastomotic integrity. The chest drain was removed after postoperative day 7.

### 2.3. Outcomes and Definitions

The primary outcome was postoperative AL, defined according to the ECCG classification [10] and diagnosed using clinical, endoscopic, and radiological criteria. Postoperative surveillance for AL was consistent throughout the study period and did not differ between the ICG and no-ICG cohorts. All patients routinely underwent upper gastrointestinal endoscopy on POD5. In the presence of earlier clinical suspicion of AL, including fever, increasing inflammatory markers such as C-reactive protein, or other suggestive clinical findings, diagnostic evaluation was anticipated. Suspected AL was assessed by upper gastrointestinal endoscopy and contrast-enhanced computed tomography, as clinically appropriate. No a priori sample-size calculation was performed, as the study included all consecutive eligible patients during the predefined study period; given the limited sample size and number of AL events, the analyses should therefore be considered exploratory and hypothesis-generating. Secondary outcomes included gastric conduit necrosis, overall postoperative morbidity (defined as the occurrence of at least one documented postoperative complication), pulmonary complications (including reintubation), infectious complications, and 90-day mortality. Within the ICG cohort, exploratory outcomes included TTF at the three predefined intraoperative timepoints, synchronous versus asynchronous fluorescence patterns between the gastroepiploic arcade and the gastric conduit, and selected hemodynamic, ventilatory, and metabolic parameters. Pathological staging was reported according to the 8th edition of the TNM classification [48]. Outpatient follow-up was scheduled at 10, 30, and 90 days after discharge to assess delayed clinical manifestations and postoperative recovery. Although the mode of data extraction differed between cohorts, the clinical surveillance and diagnostic pathway for AL remained unchanged throughout the study period.

### 2.4. Statistical Analysis

Continuous variables are reported as mean ± standard deviation (SD) or median [interquartile range (IQR)], as appropriate, for the overall between-cohort comparisons, and categorical variables as counts and percentages. Continuous variables were compared using Student’s *t*-test, and categorical variables using Fisher’s exact test, with the Fisher–Freeman–Halton extension applied to r × c contingency tables. For the primary outcome, the association between ICG implementation and postoperative anastomotic leak (AL) was expressed as an odds ratio (OR) with a Woolf logit 95% confidence interval (CI), a relative risk (RR) with a Katz logarithmic 95% CI, and an absolute risk difference (ARD) with a Newcombe hybrid-score 95% CI. Given the limited number of patients and outcome events, inference regarding the primary outcome was based primarily on the magnitude and precision of these effect estimates rather than on statistical significance.

Analyses restricted to the ICG cohort were considered exploratory and hypothesis-generating. Given the very small subgroup sizes, distributions of continuous variables were inspected and these data are presented as median [interquartile range (IQR)] and compared using the Mann–Whitney U test. Because of variable-specific missingness, the number of available observations is reported for each comparison. More than twenty intraoperative parameters were explored within the ICG cohort, and no adjustment for multiple comparisons was performed. Accordingly, all reported *p*-values from these analyses are unadjusted and should be interpreted descriptively; isolated *p*-values below 0.05 were not considered evidence of independent physiological associations with AL.

All tests were two-sided, with a nominal *p*-value < 0.05 used as the threshold for statistical significance in the primary and between-cohort analyses. Because only nine AL events occurred overall, including four within the ICG cohort, multivariable modeling, including penalized approaches such as Firth logistic regression, was considered statistically unreliable and was therefore not performed. No post hoc power calculation was performed. The limited sample size and number of outcome events resulted in substantial statistical imprecision; accordingly, the effect of ICG implementation on AL remains indeterminate, as the 95% CIs are compatible with both clinically relevant benefit and harm. Statistical analyses were performed using IBM SPSS Statistics, version 32.0 (IBM Corp., Armonk, NY, USA).

## 3. Results

### 3.1. Baseline Characteristics

Overall, 60 patients underwent Ivor-Lewis esophagectomy for malignant disease; of these, 17 (28.3%) underwent intraoperative ICG assessment of the gastric conduit and 43 (71.7%) did not. Baseline characteristics are summarized in Table 1. The two cohorts were comparable for sex, age, body mass index, smoking status, and ASA class. The ICG cohort had a higher prevalence of vascular (29.4% vs. 4.7%; *p* = 0.016) and gastrointestinal (47.1% vs. 7.0%; *p* = 0.001) comorbidity, whereas the Charlson Comorbidity Index was higher in the no-ICG cohort (6.3 vs. 4.6; *p* = 0.034).

### 3.2. Operative and Pathological Features

Operative and pathological features are reported in Table 2. Tumor localization, pathological stage, the use of neoadjuvant or perioperative chemotherapy, and operative time were comparable between cohorts. By contrast, the anastomotic technique differed significantly: a circular-stapled anastomosis (hybrid Ivor-Lewis) was performed in 5 (29.4%) patients in the ICG cohort and in 32 (74.4%) patients in the no-ICG cohort, whereas a linear-stapled anastomosis (totally minimally invasive esophagectomy) was performed in 12 (70.6%) and 11 (25.6%) patients, respectively (*p* = 0.003).

### 3.3. Short-Term Postoperative Outcomes

Short-term outcomes are shown in Table 3. The primary outcome, postoperative AL, occurred in 4 of 17 patients (23.5%) in the ICG cohort and in 5 of 43 patients (11.6%) in the no-ICG cohort. The corresponding odds ratio was 2.34 (95% CI 0.54–10.05), with an absolute risk difference of +11.9 percentage points (95% CI −7.1 to +36.5), indicating substantial uncertainty in the estimated effect. The confidence intervals were wide and compatible with both a clinically relevant reduction and a clinically relevant increase in leak risk. Overall, AL occurred in 4/23 (17.4%) patients undergoing linear-stapled anastomosis and in 5/37 (13.5%) undergoing circular-stapled anastomosis. When stratified by ICG exposure and anastomotic technique, AL occurred in 2/12 (16.7%) patients with linear-stapled and 2/5 (40.0%) patients with circular-stapled anastomosis in the ICG cohort, compared with 2/11 (18.2%) and 3/32 (9.4%), respectively, in the no-ICG cohort. According to operating surgeon, AL occurred in 7/50 (14.0%) and 2/10 (20.0%) procedures performed by surgeons 1 and 2, respectively (Table S2). Given the limited number of AL events, these data are presented descriptively without inferential subgroup comparisons.

No statistically significant differences were observed for the secondary outcomes, including gastric conduit necrosis, pulmonary complications, infectious complications, and reintubation. Overall postoperative morbidity, defined as the occurrence of at least one documented postoperative complication, was recorded in 9 of 17 patients (52.9%) in the ICG cohort and in 21 of 43 (48.8%) in the no-ICG cohort (*p* = 1.000). A single death occurred within 90 days in the no-ICG cohort (0% vs. 2.3%; *p* = 1.000).

### 3.4. Exploratory Analysis of ICG Fluorescence and Intraoperative Parameters

Within the ICG cohort, patients were further analyzed according to the occurrence of AL (4 with leak vs. 13 without leak). Intraoperative ICG dynamics and anesthesiologic parameters are detailed in Table 4. TTF did not differ between patients with and without leak in the abdominal phase (32.3 vs. 32.3 s; *p* = 0.994) or in the thoracic phase, either before (30.4 vs. 33.7 s; *p* = 0.421) or after (32.9 vs. 38.9 s; *p* = 0.438) the anastomosis. All recorded TTF values were below 60 s (range 23–46 s). The synchronous versus asynchronous pattern of fluorescence between the gastroepiploic arcade and the conduit was also similar between subgroups (synchronous in 1 of 4 patients with leak and in 4 of 13 without leak; *p* = 1.000). In no patient did ICG findings prompt a modification of the planned anastomotic site or an additional resection of the gastric conduit.

Conversely, several hemodynamic and metabolic parameters differed descriptively between subgroups. During the thoracic phase, patients who developed AL had lower PaO_2_ and PaCO_2_ and higher pH than patients without AL (Mann–Whitney U test: *p* = 0.009, *p* = 0.031, and *p* = 0.031, respectively). No corresponding differences were observed for abdominal-phase diastolic or mean arterial pressure (*p* = 0.068 and *p* = 0.133, respectively). Given the small number of assessable patients and the exploratory evaluation of multiple intraoperative variables without adjustment for multiple comparisons, these findings should be interpreted descriptively and not as evidence of independent associations with AL. Positive end-expiratory pressure and lactate did not differ between subgroups in either phase. Intraoperative inotropic support was more frequent in the AL subgroup during both the abdominal (75.0% vs. 33.3%) and thoracic (50.0% vs. 25.0%) phases, although these differences were not statistically significant.

## 4. Discussion

This study evaluated the routine implementation of qualitative intraoperative ICG fluorescence assessment of gastric conduit perfusion during esophagectomy. Given the small sample size, the limited number of AL events, and the wide confidence intervals, the available data were insufficient to determine whether ICG implementation affected the incidence of postoperative AL or overall postoperative morbidity compared with the historical no-ICG cohort. Importantly, fluorescence findings did not result in modification of the planned anastomotic site or additional conduit resection in any patient. The present study should therefore be interpreted as an evaluation of routine qualitative perfusion assessment rather than of an ICG-guided surgical strategy. No predefined fluorescence pattern or quantitative TTF threshold mandated operative intervention, and the findings cannot determine whether a standardized decision algorithm linking abnormal fluorescence to surgical modification would reduce AL. Within the ICG cohort, neither fluorescence timing nor the synchronous/asynchronous fluorescence pattern differed meaningfully according to AL occurrence, while the exploratory findings for selected intraoperative physiological variables require cautious interpretation given the small number of events and the multiplicity of comparisons.

Interpretation of the between-period comparison is further limited by temporal confounding and baseline imbalance. The ICG cohort had a higher prevalence of vascular and gastrointestinal comorbidity, whereas the Charlson Comorbidity Index was higher in the historical no-ICG cohort, indicating multidirectional differences between the two study periods. Importantly, the distribution of anastomotic techniques also differed substantially between cohorts. Linear-stapled anastomosis was introduced in October 2024, approximately five months before routine ICG implementation in March 2025, and did not replace the circular-stapled technique; both approaches continued to be used according to the operating surgeon and tumor characteristics. Descriptively, AL occurred in 17.4% (4/23) of linear-stapled and 13.5% (5/37) of circular-stapled anastomoses, while surgeon-specific rates were 14.0% (7/50) and 20.0% (2/10). These small numbers do not permit reliable inference regarding technique- or surgeon-specific effects. In addition, the learning curve associated with introduction of the linear-stapled technique represents a potential source of temporal confounding, as reported in multicenter analyses in which AL rates fell markedly only after a substantial case volume had been accrued [49]. Consequently, anastomotic technique [50], surgeon-related factors, study period, and potential concomitant changes in perioperative care, anesthesiologic management, and accumulated institutional experience cannot be reliably disentangled from ICG implementation. Given the occurrence of only nine AL events overall, these potential confounders could not be reliably addressed through multivariable adjustment. Accordingly, the unadjusted between-cohort comparison should not be interpreted as evidence of either benefit or harm attributable to ICG.

Anastomotic healing after esophagectomy is influenced by multiple interacting factors, including operative technique, conduit perfusion, nutritional and inflammatory status, smoking history, and intraoperative and postoperative oxygenation [10,51]. Gastric conduit perfusion is particularly relevant because conduit reconstruction substantially alters the native gastric vascular supply. Cigarette smoking may impair microvascular perfusion and tissue oxygenation, and both active and previous smoking have been associated with an increased risk of AL [11,17]. Strategies aimed at improving conduit perfusion have therefore received considerable attention. Meta-analytic evidence suggests that preoperative angioembolization or laparoscopic ischemic conditioning may reduce AL and, among patients who develop a leak, may decrease the need for reoperation and facilitate conservative management [18,19,20]. Reduced gastric conduit microvessel density has also been associated with higher AL rates, more major complications, and prolonged hospitalization [21]. Similarly, prospective data emphasize the importance of the right gastroepiploic artery as the dominant vascular supply of the conduit, with greater arterial length, particularly an artery-to-conduit length ratio exceeding approximately 65%, being associated with lower AL risk [15].

Within this context, ICG fluorescence imaging has been proposed as an intraoperative tool for assessing gastric conduit perfusion. Kumagai et al. proposed the “90-s rule,” recommending anastomosis in conduit areas showing fluorescence enhancement within 90 s, while considering enhancement within 60 s indicative of sufficient perfusion; areas requiring more than 90 s for enhancement were considered poorly perfused and were avoided or resected [27]. Comparable dynamic descriptors have subsequently been proposed, including analysis of gastric tube hemodynamics on fluorescence time–intensity curves, quantification of conduit blood flow speed with a suggested cut-off of 1.76 cm/s, and multicenter prospective evaluation of gastric tube blood flow [52,53,54]. A recent meta-analysis supported the potential role of ICG-based assessment in reducing AL, while emphasizing that heterogeneity in study design, selection and reporting bias, variability in leak definitions, and differences in ICG protocols may overestimate its apparent clinical effectiveness [30]. Consistently, large observational series have shown that ICG assessment modified the operative plan in only a minority of patients and did not abolish AL, which still occurred after fluorescence-guided additional conduit resection [55,56]. In a study restricted to McKeown esophagectomy with substernal reconstruction, fluorescence timing was not significantly associated with AL, although reduced fluorescence intensity was linked to a higher leak rate, potentially reflecting unrecognized conduit compression along the substernal route [35]. Other investigators have highlighted prolonged venous outflow time as a possible contributor to AL, suggesting that venous congestion may promote tissue ischemia and secondarily impair arterial inflow; however, these findings did not reach statistical significance, and no definitive outflow-time threshold has been established [36].

In the present analysis, TTF was assessed during the abdominal phase and during the thoracic phase both before and after construction of the anastomosis. All observed TTF values were below 60 s, therefore falling within the range considered indicative of adequate perfusion in the framework proposed by Kumagai et al. [27], and no meaningful differences in TTF were observed between patients with and without AL. Nevertheless, AL still occurred despite these apparently favorable fluorescence times, and its incidence remained within the range reported in previous series [9,50,57,58,59,60,61]. Similarly, the synchronous versus asynchronous fluorescence pattern between the gastroepiploic arcade and the conduit did not differ meaningfully according to AL occurrence. Taken together, these observations suggest that qualitative fluorescence timing and visual perfusion patterns, at least as applied in the present cohort and in the absence of predefined intervention criteria, may have limited discriminatory value. They should not, however, be interpreted as evidence against ICG-guided surgery, because fluorescence findings did not trigger operative modification in this study.

The predominantly qualitative and operator-dependent nature of conventional ICG imaging remains an important limitation. Preliminary studies indicate that standardized quantitative software platforms, including SERGREEN, SPY-Q, FLER, and Q-ICG, may improve diagnostic accuracy and support more objective assessment of ischemic complications by providing reproducible perfusion metrics [37,38,39]. For instance, von Kroge et al. derived parameters from fluorescence intensity curves within predefined regions of interest, including the slope of intensity, background-subtracted peak intensity, and time to slope, and proposed that these quantitative variables may contribute to AL prediction; they further suggested that a reduction in blood flow of up to approximately 32% may represent an acceptable threshold to minimize AL risk [40]. More recently, a randomized controlled trial using a quantitative fluorescence threshold to guide anastomotic-site selection reported a reduction in AL compared with visual assessment alone, supporting the potential clinical value of objective perfusion quantification [41]. Artificial intelligence may further facilitate standardized interpretation of fluorescence data [62]; however, robust clinical evidence remains insufficient, and its role in this setting has yet to be clearly defined.

Exploratory differences were also observed in selected intraoperative hemodynamic and blood gas variables. Hemodynamic stability is relevant to maintaining adequate conduit perfusion, and the need for inotropic support may reflect impaired circulatory reserve or perfusion pressure. In the present cohort, inotropic agents were administered more frequently among patients who developed AL during both the abdominal and thoracic phases. Lower diastolic and mean arterial pressures were also observed during the abdominal phase among patients who subsequently developed AL, a period during which the newly fashioned gastric conduit transitions from a multi-arterial gastric supply to a predominantly single-artery supply based on the right gastroepiploic artery. However, these blood-pressure differences did not persist on non-parametric testing and should therefore be regarded as hypothesis-generating rather than established.

Tissue oxygenation may also contribute to anastomotic healing, although the relationship between systemic oxygenation and AL has not been consistently demonstrated [63,64,65]. Differences in thoracic-phase pH, PaO_2_, and PaCO_2_ were observed between patients with and without AL in the exploratory analysis. These comparisons, however, were based on very small numbers, variable-specific missingness, and multiple unadjusted tests. Moreover, single intraoperative measurements cannot adequately characterize cumulative oxygen delivery, ventilatory exposure, or hypotension burden. Accordingly, these exploratory findings should not be interpreted as evidence of independent physiological associations with AL and do not support causal or clinically actionable conclusions.

### Limitations

This study has several limitations. First, the sample size and number of outcome events were limited, with only nine AL events overall and four within the ICG cohort. Consequently, effect estimates were imprecise, with wide confidence intervals that preclude exclusion of either a clinically relevant benefit or a clinically relevant harm associated with ICG implementation. Second, the observational and retrospective design, despite reliance on prospectively collected data, precludes causal inference. Although group assignment was determined by the timing of surgery rather than by patient-specific selection, the non-randomized before–after design remains susceptible to temporal confounding and baseline imbalance. In particular, the transition from a circular-stapled hybrid to a linear-stapled totally minimally invasive anastomosis represents an important potential confounder that could not be separated from the effect of ICG implementation. Third, the small subgroup sizes limit the reliability of secondary and exploratory comparisons. More than twenty intraoperative parameters were examined without adjustment for multiple testing, and variable-specific missingness further reduced the number of assessable patients, with several thoracic-phase comparisons based on only three patients with AL. Moreover, the abdominal-phase blood-pressure differences did not persist on non-parametric testing. Accordingly, the exploratory findings within the ICG cohort should not be interpreted as evidence of independent physiological associations with AL and require validation in larger cohorts. Fourth, because ICG findings did not alter intraoperative decision-making, this study evaluates routine qualitative fluorescence assessment rather than a genuinely ICG-guided surgical strategy and cannot determine whether intervention based on abnormal perfusion findings would reduce AL. Perfusion was assessed qualitatively without standardized quantitative software, potentially limiting the discriminatory capacity and reproducibility of ICG imaging. TTF was based on visual identification of fluorescence onset and was therefore observer-dependent; moreover, residual fluorescence from repeated ICG administrations may have influenced subsequent assessments. Fifth, the mode of complication data extraction differed between cohorts. Complications were recorded through a structured checklist in the ICG cohort and through narrative clinical fields in the historical cohort, potentially resulting in more complete ascertainment of minor events in the former; overall morbidity should therefore be interpreted conservatively. Complications were classified according to ECCG definitions rather than by generic severity grading. Detailed characteristics of individual AL events are provided in Supplementary Table S2; however, given the occurrence of only nine leaks, these data are descriptive and do not permit reliable comparisons of leak severity or management between cohorts. With a single death within 90 days across the entire cohort, the mortality comparison is uninformative, and longer-term survival was not assessed. Finally, the single-center design and limited number of operating surgeons may restrict the external generalizability of these findings.

## 5. Conclusions

In this small single-center before–after study, the available data were insufficient to determine whether routine implementation of intraoperative ICG fluorescence assessment affects the incidence of AL. Fluorescence findings did not result in modification of the planned surgical strategy, and therefore these findings should not be interpreted as an assessment of the effectiveness of a truly ICG-guided intervention. Larger prospective studies evaluating quantitative, decision-guided ICG protocols are warranted.

## Figures and Tables

**Table 1:** Preoperative clinical characteristics of the study cohorts.

Characteristic	ICG (n = 17)	No-ICG (n = 43)	*p*-Value
Sex, n (%)			0.132
Male	14 (82.4)	41 (95.3)	
Female	3 (17.6)	2 (4.7)	
Age, years	66.8 ± 9.4	65.2 ± 13.0	0.660
Body mass index, kg/m^2^	25.9 ± 5.6	24.5 ± 3.5	0.362
Comorbidity, n (%)			
Hypertension	9 (52.9)	17 (39.5)	0.396
Diabetes	3 (17.6)	9 (20.9)	1.000
Respiratory disease	3 (17.6)	6 (14.0)	0.704
Vascular disease	5 (29.4)	2 (4.7)	**0.016**
Cardiac disease	5 (29.4)	6 (14.0)	0.265
Gastrointestinal disease	8 (47.1)	3 (7.0)	**0.001**
Active smoking, n (%)	3 (17.6)	16 (37.2)	0.219
Serum albumin concentration (g/dL)	3.8 ± 0.5	3.9 ± 0.4	0.81
ASA class, n (%)			0.884
II	7 (41.2)	15 (34.9)	
III	10 (58.8)	26 (60.5)	
IV	0 (0.0)	2 (4.7)	
ASA class > II, n (%)	10 (58.8)	28 (65.1)	0.768
Charlson Comorbidity Index	4.6 ± 1.6	6.3 ± 3.0	**0.034**

Data are mean ± standard deviation for continuous variables and n (%) for categorical variables; percentages refer to the total number of patients in each cohort. Continuous variables were compared using Student’s *t*-test and categorical variables using Fisher’s exact test (Fisher–Freeman–Halton extension for r × c tables). ASA, American Society of Anesthesiologists; ICG, indocyanine green. Bold *p*-values denote statistical significance (*p* < 0.05).

**Table 2:** Operative and pathological features of the study cohorts.

Feature	ICG (n = 17)	No-ICG (n = 43)	*p*-Value
Tumor localization, n (%)			1.000
Middle esophagus	2 (11.8)	6 (14.0)	
Lower esophagus	8 (47.1)	19 (44.2)	
Esophagogastric junction	7 (41.2)	18 (41.9)	
Type of anastomosis, n (%)			**0.003**
Circular (hybrid Ivor-Lewis)	5 (29.4)	32 (74.4)	
Linear (totally minimally invasive)	12 (70.6)	11 (25.6)	
Operative time, min	359.5 ± 70.9	348.0 ± 59.2	0.551
Pathological stage (p or yp), n (%)			0.983
0	0 (0.0)	1 (2.3)	
I	3 (17.6)	8 (18.6)	
II	7 (41.2)	19 (44.2)	
III	4 (23.5)	9 (20.9)	
IVa	3 (17.6)	6 (14.0)	
Perioperative chemotherapy (FLOT), n (%)	10 (58.8)	27 (62.8)	0.777

Data are mean ± standard deviation for continuous variables and n (%) for categorical variables; percentages refer to the total number of patients in each cohort. Pathological stage is reported according to the 8th edition of the TNM classification. Continuous variables were compared using Student’s *t*-test and categorical variables using Fisher’s exact test (Fisher–Freeman–Halton extension for r × c tables). ICG, indocyanine green. Bold *p*-values denote statistical significance (*p* < 0.05).

**Table 3:** Short-term postoperative outcomes.

Outcome	ICG (n = 17)	No-ICG (n = 43)	*p*-Value
Anastomotic leak, n (%)	4 (23.5)	5 (11.6)	0.256
Gastric conduit necrosis, n (%)	1 (5.9)	0 (0.0)	0.283
Other gastrointestinal complications, n (%)	1 (5.9)	0 (0.0)	0.283
Pneumonia, n (%)	1 (5.9)	2 (4.7)	1.000
Pleural effusion, n (%)	2 (11.8)	5 (11.6)	1.000
Pneumothorax, n (%)	2 (11.8)	2 (4.7)	0.317
Respiratory failure, n (%)	2 (11.8)	1 (2.3)	0.191
Acute respiratory distress syndrome, n (%)	3 (17.6)	1 (2.3)	0.065
Tracheobronchial injury, n (%)	1 (5.9)	1 (2.3)	0.490
Chest tube > 10 days for air leak, n (%)	0 (0.0)	1 (2.3)	1.000
Wound infection, n (%)	0 (0.0)	1 (2.3)	1.000
Central line-associated bloodstream infection, n (%)	1 (5.9)	0 (0.0)	0.283
Organ/space surgical site infection, n (%)	0 (0.0)	0 (0.0)	1.000
Sepsis, n (%)	1 (5.9)	0 (0.0)	0.283
Other infections requiring antibiotics, n (%)	2 (11.8)	2 (4.7)	0.317
Reintubation, n (%)	3 (17.6)	1 (2.3)	0.065
Overall postoperative morbidity, n (%) ^a^	9 (52.9)	21 (48.8)	1.000
90-day mortality, n (%)	0 (0.0)	1 (2.3)	1.000

Data are n (%); percentages refer to the total number of patients in each cohort. Categorical variables were compared using Fisher’s exact test. ^a^ Overall postoperative morbidity was defined as the occurrence of at least one documented postoperative complication. ICG, indocyanine green.

**Table 4:** Intraoperative ICG fluorescence dynamics and anesthesiologic parameters within the ICG cohort, stratified by the occurrence of anastomotic leak.

Parameter	Leak (n = 4)	No Leak (n = 13)	*p*-Value
Abdominal TTF, s	32.1 [27.3–37.1]	31 [23–40.1]	0.994
Thoracic TTF, pre-anastomosis, s	33.4 [27.7–34.6]	34.4 [28.7–38.9]	0.421
Thoracic TTF, post-anastomosis, s	34 [29–37.4]	39 [32.6–46]	0.438
Abdominal systolic blood pressure, mmHg	100 [82.8–116]	124 [102–132]	0.090
Abdominal diastolic blood pressure, mmHg	53 [49–59]	65 [61–79]	**0.041**
Abdominal mean arterial pressure, mmHg	69 [60–78]	84 [75–95.7]	**0.043**
Thoracic systolic blood pressure, mmHg	122 [120–128]	113 [98–125]	0.270
Thoracic diastolic blood pressure, mmHg	70 [62–73]	61 [56.3–64.8]	0.601
Thoracic mean arterial pressure, mmHg	83.8 [57–91]	78.8 [69–84]	0.382
Heart rate during ICG, abdomen, bpm	67 [60–73]	61 [56–68]	0.626
Heart rate during ICG, thorax, bpm	52 [50–57]	57 [55–72]	0.264
PEEP during ICG, abdomen, cmH_2_O	7.5 [4.8–10]	5 [5–7]	0.298
PEEP during ICG, thorax, cmH_2_O	7 [6–9.5]	6 [5–7]	0.213
PaO_2_, abdomen, mmHg	114 [104–135]	131 [104–152]	0.637
PaO_2_, thorax, mmHg	66 [60–69]	123 [84–148]	**0.049**
PaCO_2_, abdomen, mmHg	53.4 [43–64]	49 [40–55]	0.438
PaCO_2_, thorax, mmHg	37.4 [37–40]	48 [42–52]	**0.039**
pH, abdomen	7.3 [7.2–7.4]	7.3 [7.3–7.3]	0.563
pH, thorax	7.4 [7.4–7.4]	7.3 [7.2–7.3]	**0.023**
Lactate, abdomen, mmol/L	1 [0.9–1.2]	1.3 [0.9–1.5]	0.607
Lactate, thorax, mmol/L	1.3 [1.1–1.8]	1.3 [0.9–1.9]	0.803
Inotropic drugs, abdomen, n (%) ^a^	3 (75.0)	4 (33.3)	0.262
Inotropic drugs, thorax, n (%) ^a^	2 (50.0)	3 (25.0)	0.547
ICG pattern (arcade vs. conduit), n (%)			1.000
Synchronous	1 (25.0)	4 (30.8)	
Asynchronous	3 (75.0)	9 (69.2)	

Data are median [interquartile range] for continuous variables and n (%) for categorical variables. Continuous variables were compared using the Mann–Whitney U test and categorical variables using Fisher’s exact test. Because of incomplete intraoperative records, the number of assessable patients varied by parameter: 4 and 13 for abdominal time to fluorescence; 3 and 13 for pre-anastomotic and 3 and 7 for post-anastomotic thoracic time to fluorescence; 4 and 12 for abdominal-phase haemodynamic, ventilatory and blood gas variables; and 3 and 12 for the corresponding thoracic-phase variables. ^a^ Denominators for inotropic drug administration are 4 and 12. bpm, beats per minute; ICG, indocyanine green; PaCO_2_, arterial partial pressure of carbon dioxide; PaO_2_, arterial partial pressure of oxygen; PEEP, positive end-expiratory pressure; TTF, time to fluorescence. Bold *p*-values indicate nominal *p* < 0.05; all exploratory *p*-values are unadjusted and should be interpreted descriptively.

## Data Availability

The data presented in this study are available on request from the corresponding author. The data are not publicly available because of privacy restrictions concerning patient information.

## Supplementary Materials

The following supporting information can be downloaded at: https://www.mdpi.com/article/10.3390/jcm15176827/s1. Video S1: Intraoperative ICG fluorescence assessment of the gastric conduit during the abdominal phase; Video S2: Intraoperative ICG fluorescence assessment of the gastric conduit during the thoracic phase (hybrid Ivor-Lewis); Table S1: STROBE checklist for cohort studies. Table S2: Individual clinical characteristics, diagnosis, management, and outcomes of patients with anastomotic leak.

## Abbreviations

The following abbreviations are used in this manuscript:

AL	anastomotic leak
ARD	absolute risk difference
ASA	American Society of Anesthesiologists
CI	confidence interval
ECCG	Esophagectomy Complications Consensus Group
ICG	indocyanine green
OR	odds ratio
PaCO_2_	arterial partial pressure of carbon dioxide
PaO_2_	arterial partial pressure of oxygen
PEEP	positive end-expiratory pressure
RR	relative risk
STROBE	Strengthening the Reporting of Observational Studies in Epidemiology
TNM	tumor–node–metastasis
TTF	time to fluorescence
