# Supplementary material for: Routine Intraoperative ICG Perfusion Assessment and Anastomotic Leak After Esophagectomy: A Before–After Cohort Study
Source: J Clin Med. 2026 Sep 3;15(17):6827. doi: 10.3390/jcm15176827 (PMC13566163; doi:10.3390/jcm15176827)
Supplement: Supplementary file 1 [file jcm-15-06827-s001.zip › jcm-4527616-supplementary.pdf]

Supplementary Materials

Contents

Video S1. Intraoperative ICG fluorescence assessment of the gastric conduit during the abdominal phase.

Video S2. Intraoperative ICG fluorescence assessment of the gastric conduit during the thoracic phase (hybrid Ivor-Lewis).

Table S1. STROBE checklist for cohort studies.

Table S2. Individual clinical characteristics, diagnosis, management, and outcomes of patients with anastomotic leak.

Video S1. Abdominal-phase ICG fluorescence assessment

Near-infrared fluorescence recording obtained during the abdominal phase of a totally minimally invasive Ivor-Lewis esophagectomy, after intravenous administration of 2 mL of indocyanine green solution (25 mg diluted in 10 mL of saline) through a peripheral venous catheter. The recording illustrates progressive opacification of the gastroepiploic arcade and of the gastric conduit and the assessment of time to fluorescence at the predicted anastomotic site. Duration: 14 s. No patient-identifiable information is displayed.

Video S2. Thoracic-phase ICG fluorescence assessment

Near-infrared fluorescence recording obtained during the thoracic phase of a hybrid Ivor-Lewis esophagectomy, after intravenous administration of 2 mL of indocyanine green solution (25 mg diluted in 10 mL of saline) through the same peripheral venous catheter, before construction of the circular-stapled intrathoracic esophagogastric anastomosis. Duration: 14 s. No patient-identifiable information is displayed.

Table S1. STROBE checklist for cohort studies

Reporting of this observational study follows the STROBE recommendations. For each item, the location within the manuscript where the item is addressed is indicated.

Item	Section	Recommendation	Location in the manuscript
1a	Title and abstract	Study design indicated in the title and abstract	Title; Abstract
1b	Title and abstract	Balanced and informative summary of what was done and found	Abstract
2	Introduction	Scientific background and rationale for the investigation	Section 1
3	Introduction	Prespecified objectives, including any prespecified hypotheses	Section 1 (final paragraph)
4	Methods	Key elements of study design presented early in the paper	Section 2.1; Figure 1
5	Methods	Setting, locations and relevant dates of recruitment, exposure, follow-up and data collection	Section 2.1; Figure 1
6a	Methods	Eligibility criteria, sources and methods of participant selection; methods of follow-up	Section 2.1; Section 2.2
6b	Methods	For matched studies, matching criteria and number of exposed and unexposed participants	Not applicable (unmatched before–after design)
7	Methods	Clear definition of outcomes, exposures, predictors, potential confounders and effect modifiers	Sections 2.1–2.3

Item	Section	Recommendation	Location in the manuscript
8	Methods	Sources of data and details of methods of assessment for each variable of interest	Sections 2.2–2.3
9	Methods	Efforts to address potential sources of bias	Sections 2.1, 2.4; Section 4.1
10	Methods	Explanation of how the study size was arrived at	Section 2.1 (all consecutive eligible patients in the study period); Section 4.1
11	Methods	Handling of quantitative variables and grouping choices	Sections 2.3–2.4
12a	Methods	Statistical methods, including those used to control for confounding	Section 2.4
12b	Methods	Methods used to examine subgroups and interactions	Section 2.4 (exploratory within-ICG analyses)
12c	Methods	How missing data were addressed	Section 2.4; Table 4 footnote
12d	Methods	How loss to follow-up was addressed	Section 2.3 (follow-up at 10, 30 and 90 days)
12e	Methods	Sensitivity analyses	Not performed; justified in Sections 2.4 and 4.1
13a	Results	Numbers of individuals at each stage of the study	Section 3.1; Figure 1
13b	Results	Reasons for non-participation at each stage	Section 2.1 (consecutive patients; no exclusions after eligibility)
13c	Results	Flow diagram	Figure 1
14a	Results	Characteristics of study participants and information on exposures and potential confounders	Section 3.1; Tables 1–2
14b	Results	Number of participants with missing data for each variable of interest	Table 4 footnote
14c	Results	Follow-up time	Section 2.3
15	Results	Numbers of outcome events or summary measures over time	Section 3.3; Table 3
16a	Results	Unadjusted and, where applicable, confounder-adjusted estimates with 95% confidence intervals	Section 3.3 (unadjusted OR, RR and ARD with 95% CI; adjusted estimates not calculable, see Section 2.4)
16b	Results	Category boundaries when continuous variables were categorised	Not applicable
16c	Results	Translation of relative risk into absolute risk for a meaningful time period	Section 3.3 (absolute risk difference reported)
17	Results	Other analyses performed (subgroups, interactions, sensitivity analyses)	Section 3.4; Table 4
18	Discussion	Key results summarised with reference to study objectives	Section 4 (first paragraph)
19	Discussion	Limitations, including sources of potential bias or imprecision	Section 4.1
20	Discussion	Cautious overall interpretation considering objectives, limitations, multiplicity and other evidence	Sections 4, 4.1 and 5
21	Discussion	Generalisability (external validity) of the results	Section 4.1 (final sentence)
22	Other information	Source of funding and role of the funders	Funding statement

STROBE, Strengthening the Reporting of Observational Studies in Epidemiology. The checklist is available at https://www.strobe-statement.org.

Patient	ICG assessment	POD at AL diagnosis	Anastomotic technique	ECCG type	Diagnostic modality	Gastric conduit necrosis	Endoscopic intervention	Surgical intervention	ICU admission	Clinical outcome
Pt No. 1	N	6	Circular	II	Endoscopy CT scan	N	Stent	N	N	Healing
Pt No. 2	N	5	Linear	I	Endoscopy	N	Nasojejunal feeding tube	N	N	Healing
Pt No. 3	N	7	Circular	II	Endoscopy	N	EsoSponge	Jejunostomy	Y	Healing
Pt No. 4	N	9	Linear	II	Endoscopy CT scan	N	EsoSponge	N	N	Healing
Pt No. 5	N	5	Circular	II	Endoscopy	N	Stent	N	N	Healing
Pt No. 6	Y	3	Circular	III	Endoscopy CT scan	Y	N	Cervical esophagostomy	Y	Death
Pt No. 7	Y	4	Linear	II	Endoscopy	N	OTSC	N	N	Healing
Pt No. 8	Y	5	Linear	II	Endoscopy	N	EsoSponge	N	N	Healing
Pt No. 9	Y	5	Circular	II	Endoscopy	N	EsoSponge	N	N	Healing

Supplementary Table S2. Abbreviations: AL, anastomotic leak; ECCG, Esophagectomy Complications Consensus Group; ICG, indocyanine green; ICU, intensive care unit; POD, postoperative day.
